# Supplementary material for: Unraveling radiation damage and healing mechanisms in halide perovskites using energy-tuned dual irradiation dosing
Source: Nat Commun. 2024 Jan 24;15:696. doi: 10.1038/s41467-024-44876-1 (PMC10810841; doi:10.1038/s41467-024-44876-1)
Supplement: Supplementary file 1 — Supplementary Information [file 41467_2024_44876_MOESM1_ESM.pdf]

Supplementary Information

Unraveling radiation damage and healing mechanisms in halide perovskites using energy-tuned dual irradiation dosing

Ahmad R. Kirmani,[1, 2,*] Todd A. Byers,[3] Zhenyi Ni,[4] Kaitlyn VanSant,[1,5] Darshpreet K. Saini,[3] Rebecca Scheidt,[1] Xiaopeng Zheng,[1] Tatchen Buh Kum,[2] Ian R. Sellers,[6] Lyndsey McMillon-Brown,[5] Jinsong Huang,[4] Bibhudutta Rout,[3] Joseph M. Luther[1*]

[1]National Renewable Energy Laboratory (NREL), CO 80215, USA
[2]School of Chemistry and Materials Science, Rochester Institute of Technology, NY 14623, USA
[3]Department of Physics, University of North Texas, TX 76203, USA
[4]Department of Applied Physical Sciences, University of North Carolina, Chapel Hill, NC 27599, USA
[5]NASA Glenn Research Center, Cleveland, OH 44135, USA
[6]Homer L. Dodge Department of Physics and Astronomy, University of Oklahoma, OK 73019, USA

*ahmad.kirmani@rit.edu; joey.luther@nrel.gov

Table of Contents

Supplementary Figure 1. Comparison of NIELs for alpha particles, protons, and electrons. 3
Supplementary Figure 2. IEL/NIEL ratio as a function of proton energy. 4
Supplementary Figure 3. X-SEM of *NIP* device. 5
Supplementary Figure 4. Defect densities created by 0.06 MeV and 1.0 MeV protons. 6
Supplementary Figure 5. Cumulative NIEL and IEL in *NIP* devices. 7
Supplementary Figure 6. Remaining factors for irradiated *NIP* devices. 8
Supplementary Figure 7. Elemental vacancy profiles for 1.0 MeV proton irradiation. 9
Supplementary Figure 8. NIEL and IEL profiles for *PIN* devices. 10
Supplementary Figure 9. Cumulative NIEL and IEL in *PIN* devices. 11
Supplementary Figure 10. X-SEM of *PIN* device. 12
Supplementary Figure 11. TRPL data. 13
Supplementary Table 1. 13
Supplementary Figure 12. NIEL and IEL curves for 'normalized' vs 'non-normalized' scenarios. 14
Supplementary Figure 13. Cumulative NIEL and IEL for 'normalized' and 'not normalized' scenarios. 15
Supplementary Figure 14. PCE remaining factors with increasing NIEL. 16
Supplementary Figure 15. PCE remaining factors for dark aging and light soaking experiments. 17
Supplementary Figure 16. Remaining factors for thermal conditioning experiments. 18
Supplementary Figure 17. SRIM simulations for 0.01 MeV, 0.03 MeV, 0.06 MeV proton irradiation. 19
Supplementary Figure 18. Dual-dose experiments on device architecture employing a modified charge transport layer. 20
Supplementary Table 2. 21
Supplementary Note 1 21
Supplementary Figure 19: SRIM vacancy profiles for 1 μm SiO_X PIN PSCs. 22
Supplementary Figure 20: Dual-dose experiments on 1 μm-SiO_X *PIN* PSCs. 23
Supplementary Note 2 24
Supplementary References 25

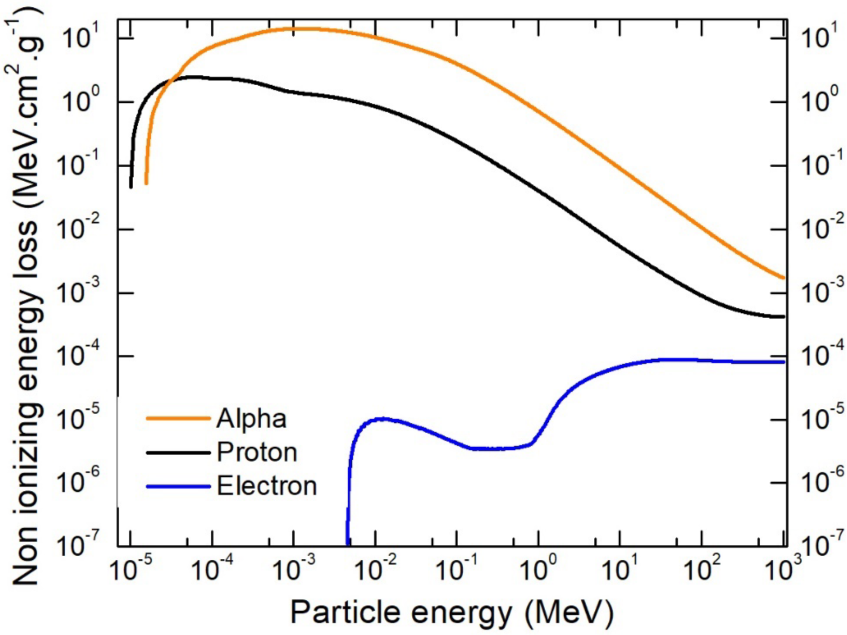

Supplementary Figure 1. Comparison of NIELs for alpha particles, protons, and electrons. Comparison of NIELs for alpha particles, protons, and electrons incident on PSCs as a function of the incident particle energy.

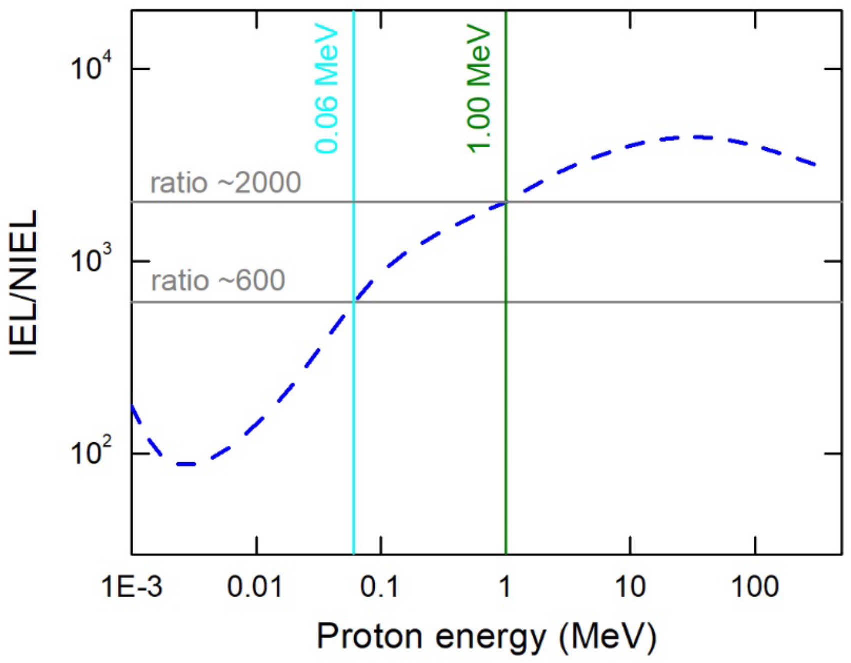

Supplementary Figure 2. IEL/NIEL ratio as a function of proton energy.
IEL/NIEL ratio as a function of proton energy calculated using SR-NIEL.[1,2] Horizontal gray lines highlight that the ratio increases from ~600 to ~2000 from 0.06 MeV to 1.00 MeV proton energy.

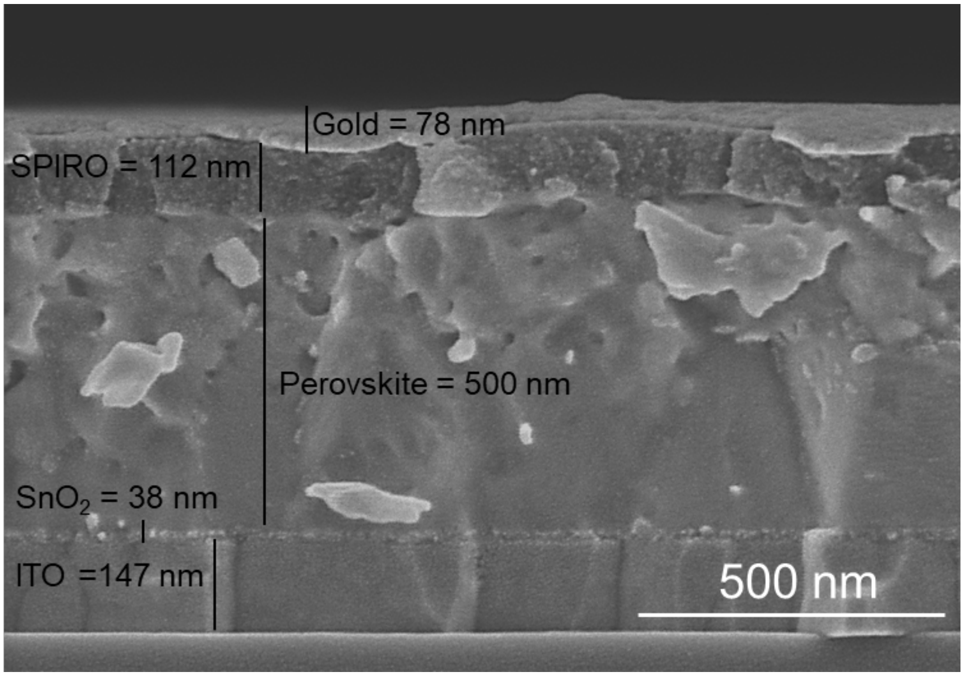

Supplementary Figure 3. X-SEM of *NIP* device.
X-SEM image of a representative *NIP* solar cell used in this study.

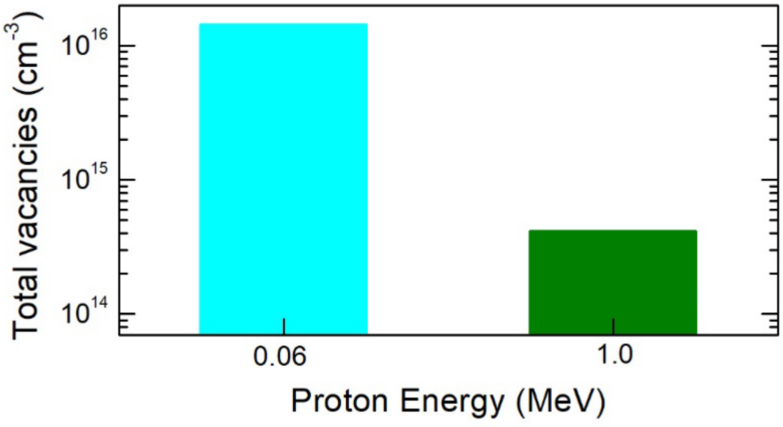

Supplementary Figure 4. Defect densities created by 0.06 MeV and 1.0 MeV protons.

Defect density in the perovskite device created by normally-incident protons with energy of 0.06 MeV (cyan) and 1.0 MeV (green) and $1x10^{13}$ cm^{-2} fluence. A device thickness of 500 nm was considered for converting the total number of defects from SRIM into defect density.

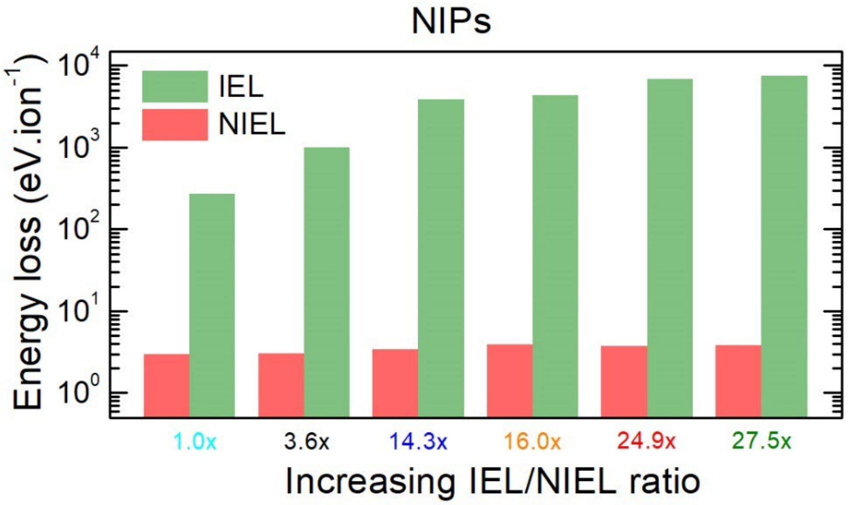

Supplementary Figure 5. Cumulative NIEL and IEL in *NIP* devices.
Cumulative NIEL (red) and IEL (green) within *NIP* devices.

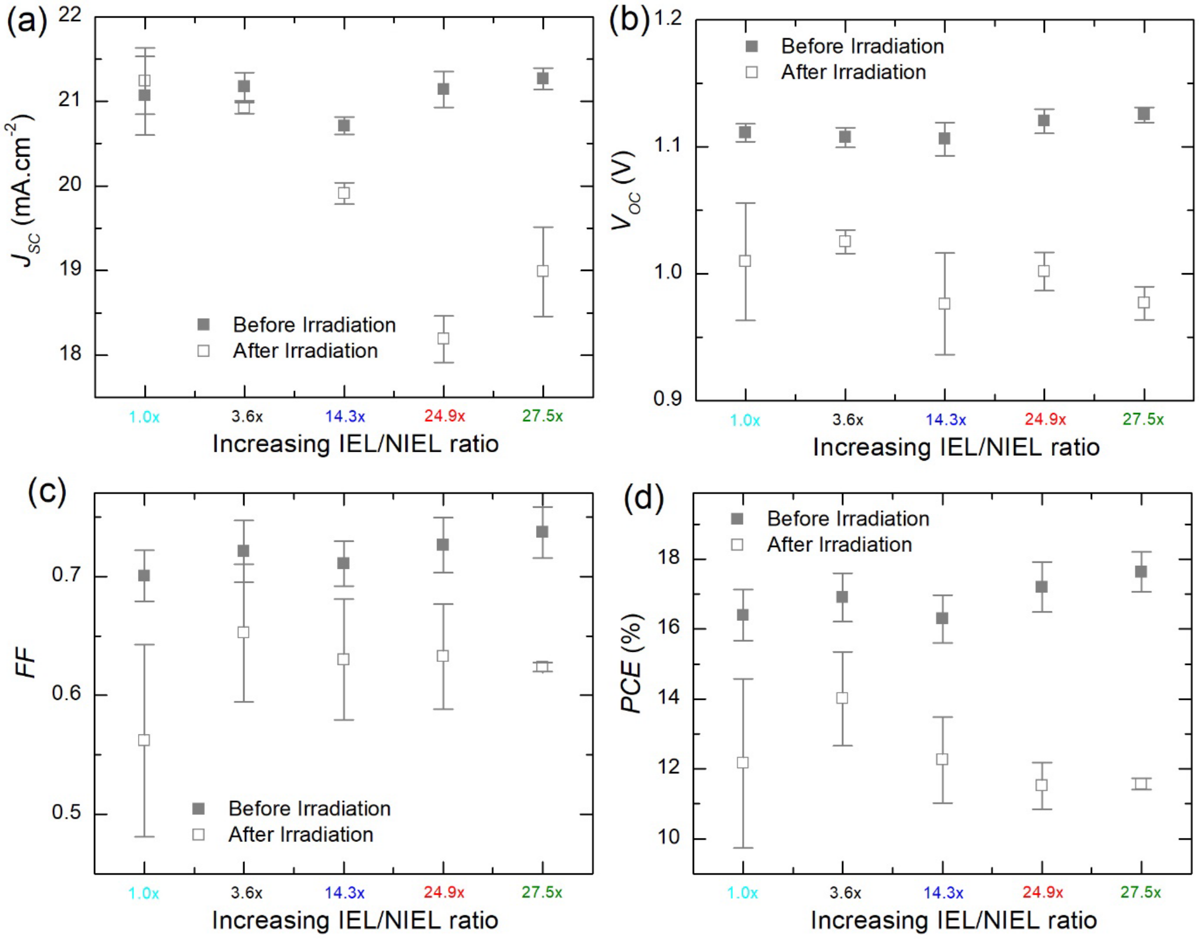

Supplementary Figure 6. Remaining factors for irradiated *NIP* devices.

(a) J_{SC}, (b) V_{OC}, (c) *FF*, and (d) *PCE* of *NIP* solar cells before (full squares) and after (empty squares) irradiation for various IEL scenarios. Parameters are averaged over 4-5 devices. Error bars represent standard deviation.

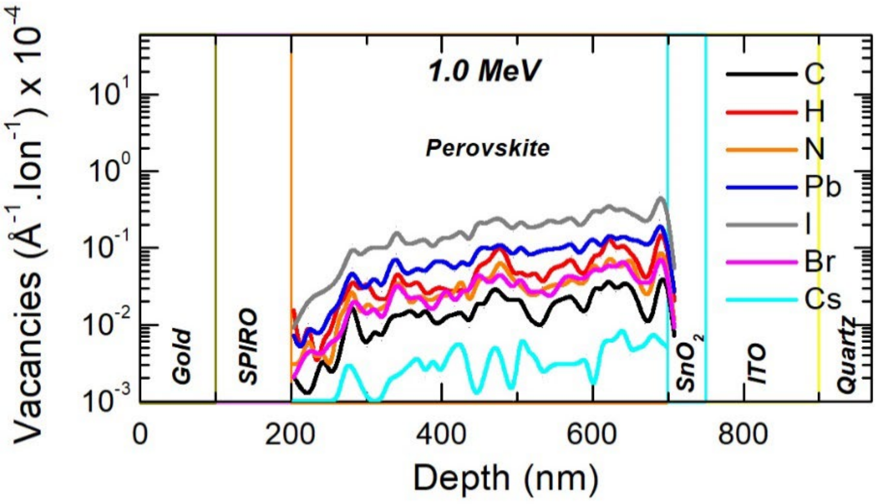

Supplementary Figure 7. Elemental vacancy profiles for 1.0 MeV proton irradiation.
SRIM simulations showing elemental vacancy profiles within the perovskite active layer for 1.0 MeV protons. I (grey) and Pb (blue) are the most displaced elements.

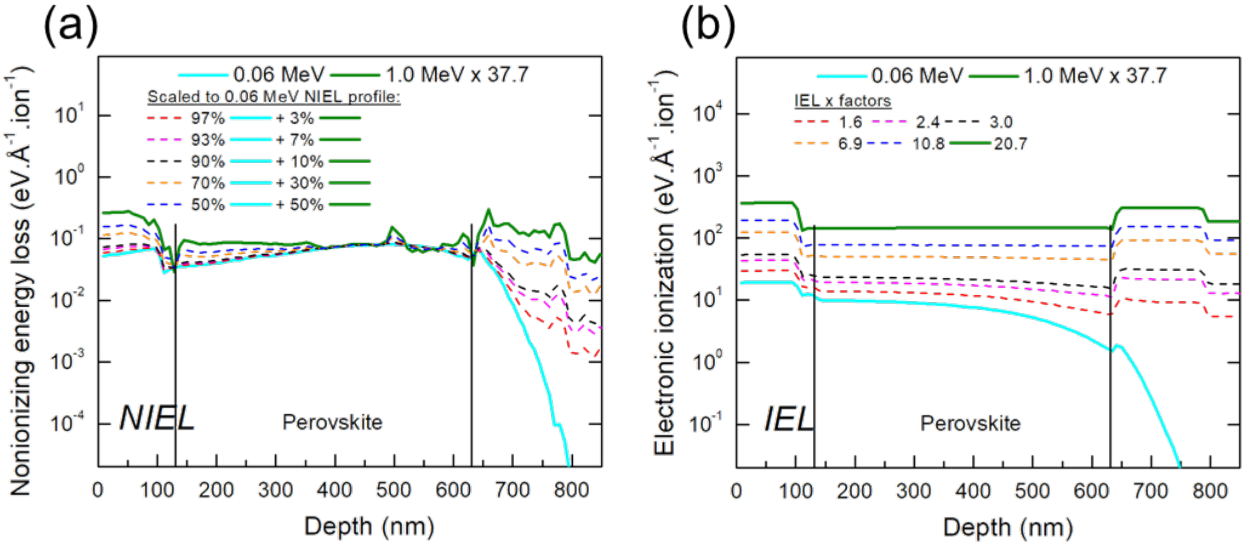

Supplementary Figure 8. NIEL and IEL profiles for *PIN* devices.

(a) NIEL, and (b) IEL profiles for the various irradiation scenarios for *PIN* solar cells.

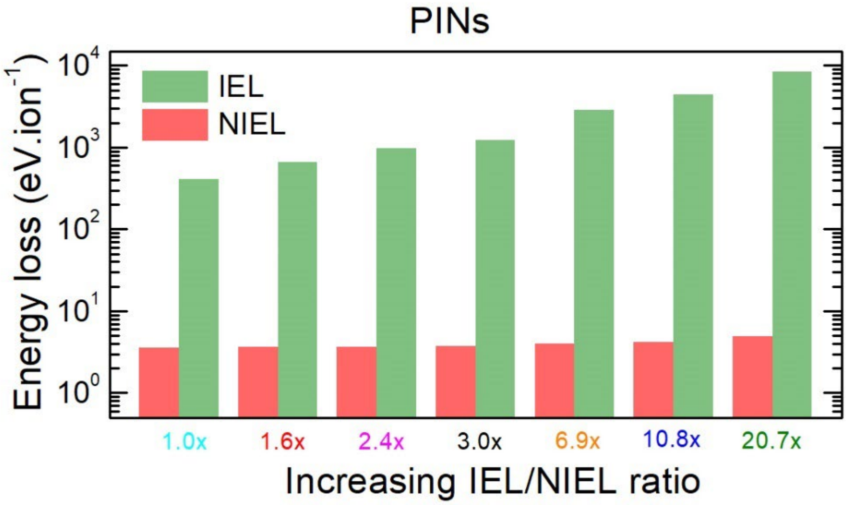

Supplementary Figure 9. Cumulative NIEL and IEL in *PIN* devices.
Cumulative NIEL (red) and IEL (green) within *PIN* devices.

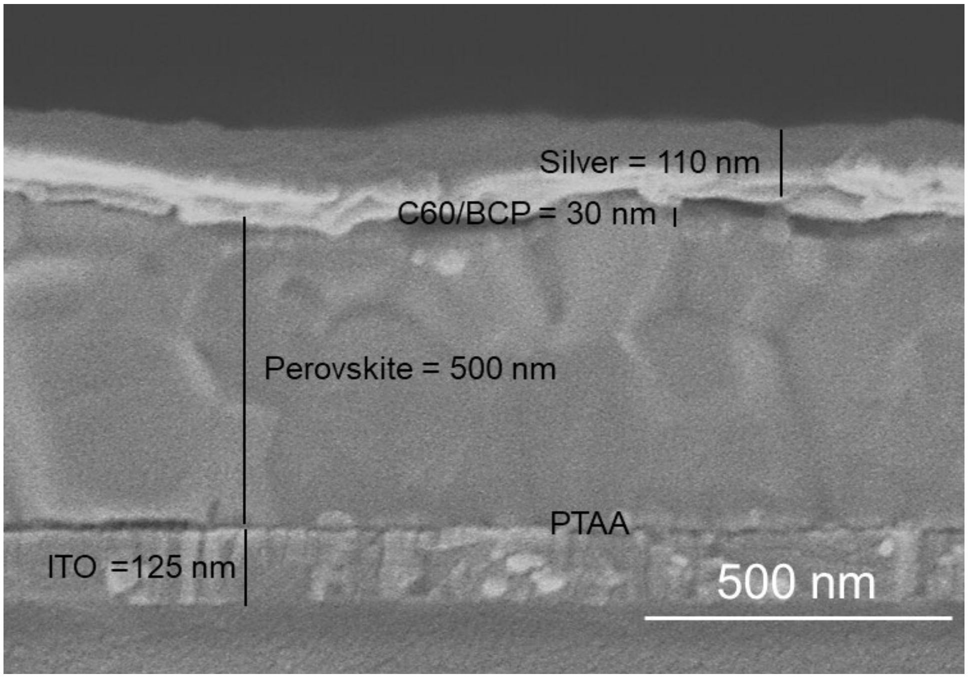

Supplementary Figure 10. X-SEM of *PIN* device.
X-SEM image of a representative *PIN* solar cell used in this study.

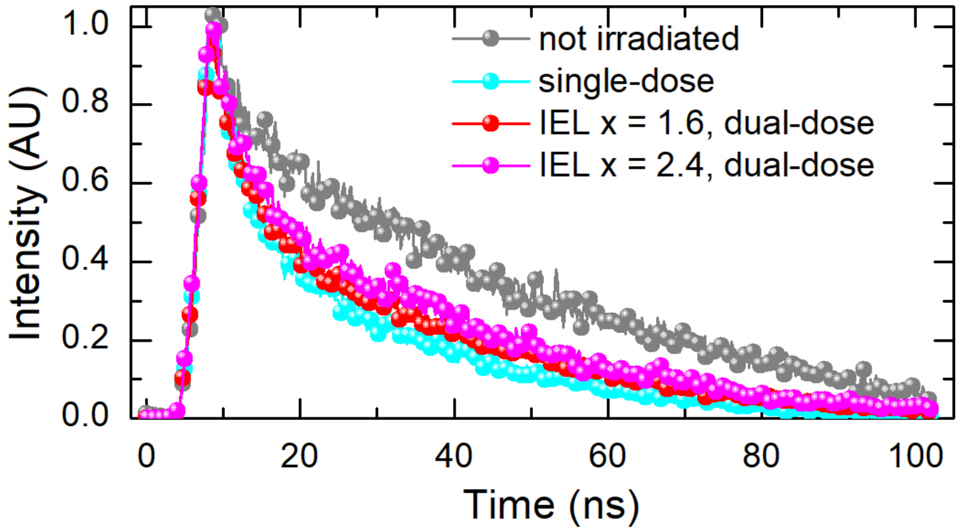

Supplementary Figure 11. TRPL data.
TRPL plots and fitting curves on device stacks without metal electrode.

Supplementary Table 1.
Carrier decay times and decay amplitudes obtained from a biexponential fit to the TRPL curves.

Device	A_1	τ_1 (ns)	A_2	τ_2 (ns)
Not irradiated	0.22	2.3 ± 0.0	0.89	53.9 ± 2.0
Single dose	0.46	3.5 ± 0.1	0.55	28.1 ± 0.6
IEL x = 1.6	0.44	3.9 ± 0.0	0.58	36.8 ± 0.8
IEL x = 2.4	0.38	3.7 ± 0.1	0.73	46.8 ± 1.3

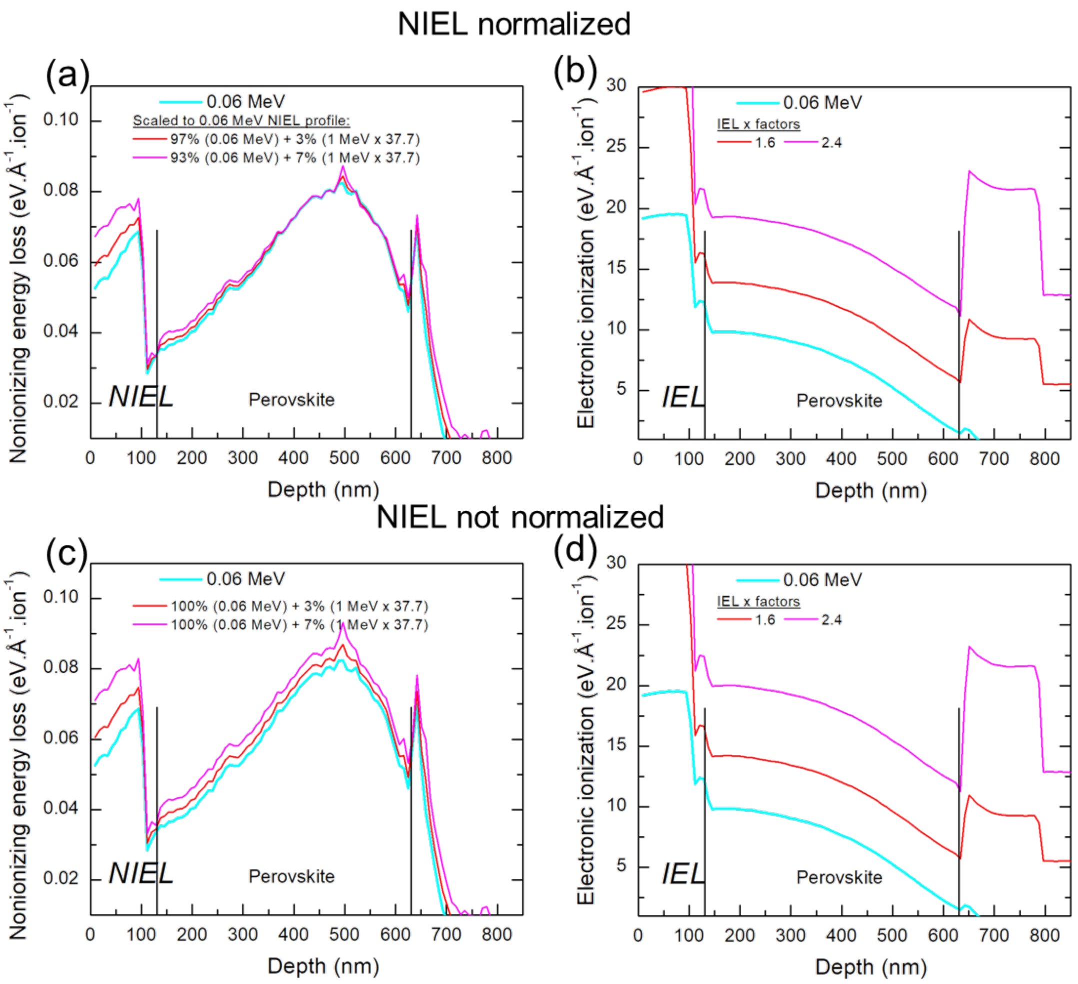

Supplementary Figure 12. NIEL and IEL curves for 'normalized' vs 'non-normalized' scenarios.

NIEL and IEL curves for the (a), (b) 'NIEL normalized', and (c), (d) 'NIEL not normalized' cases.

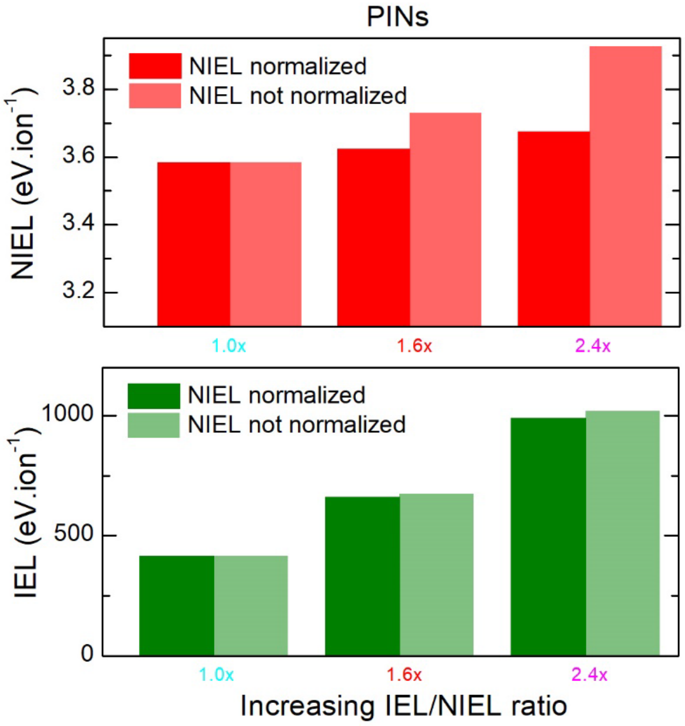

Supplementary Figure 13. Cumulative NIEL and IEL for 'normalized' and 'not normalized' scenarios.

Cumulative NIEL (top) and IEL (bottom) for the 'normalized' and 'not normalized' cases.

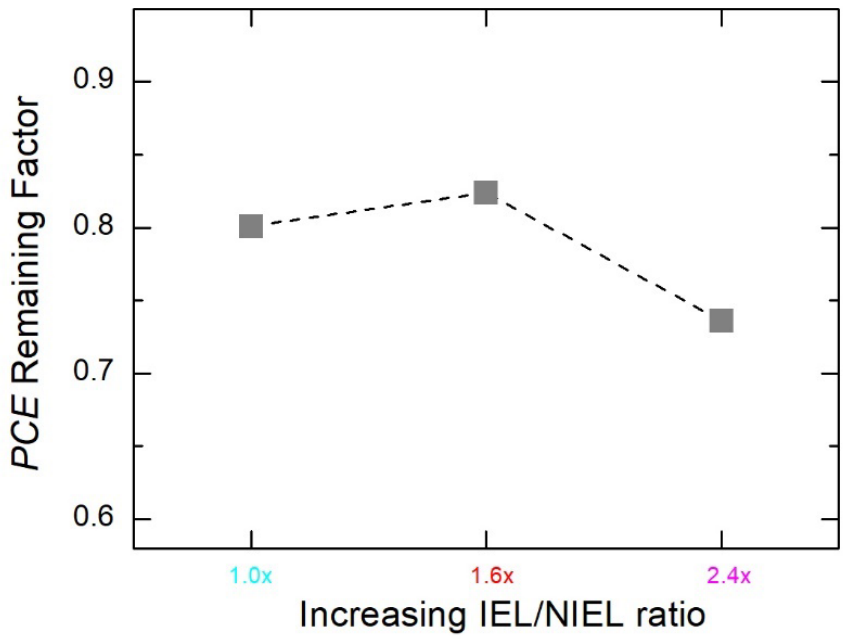

Supplementary Figure 14. PCE remaining factors with increasing NIEL.
PCE remaining factors for dual dose experiments with an increasing NIEL dose.

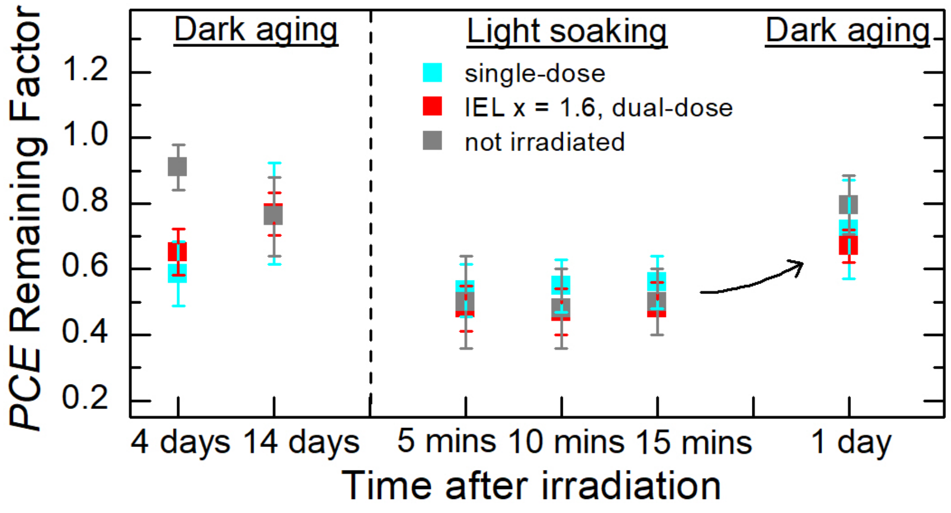

Supplementary Figure 15. PCE remaining factors for dark aging and light soaking experiments.

PCE remaining factors for *PIN* solar cells for dark aging and light soaking experiments. Parameters are averaged over 4-5 devices. Error bars represent standard deviation.

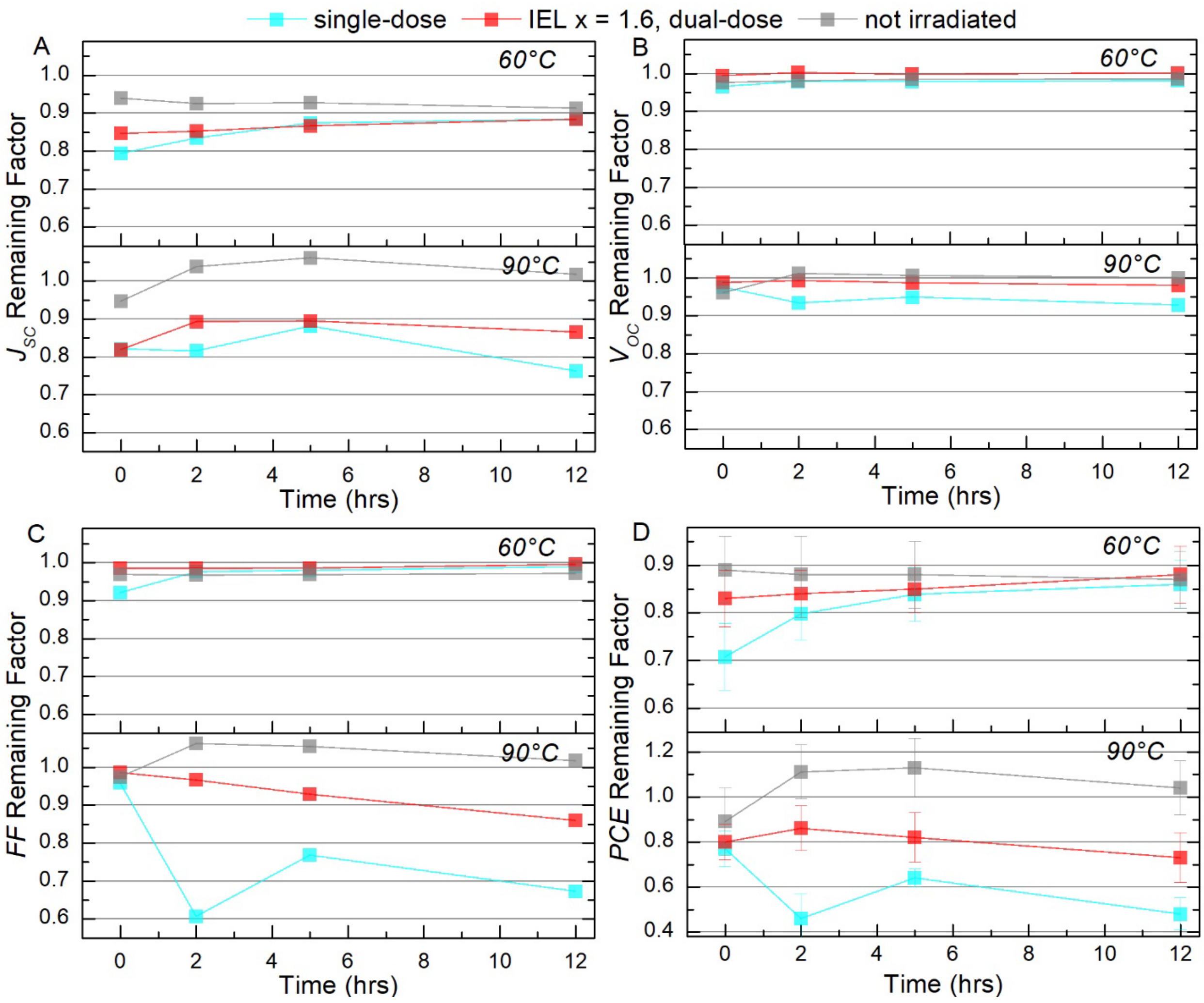

Supplementary Figure 16. Remaining factors for thermal conditioning experiments.

Remaining factors of *PIN* solar cells for the temperature annealing experiments at 60°C and 90°C. (a) J_{SC}, (b) V_{OC}, (c) *FF*, and (d) *PCE* remaining factors are shown, and parameters are averaged over 4-5 devices. Error bars represent standard deviation.

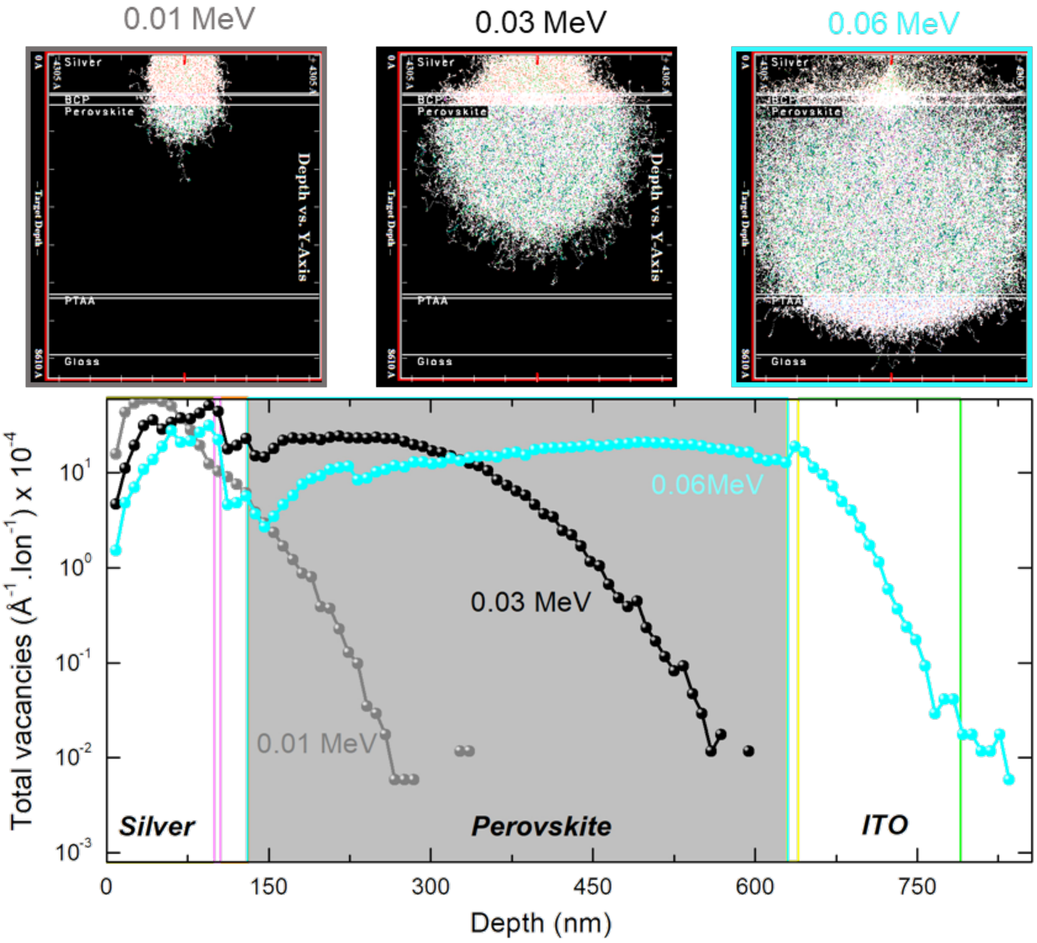

Supplementary Figure 17. SRIM simulations for 0.01 MeV, 0.03 MeV, 0.06 MeV proton irradiation.

(Top). SRIM simulations showing proton straggling for 0.01 MeV, 0.03 MeV, and 0.06 MeV protons incident on *PIN* solar cells. (Bottom). Corresponding vacancy profiles. Shaded region highlights the perovskite active layer.

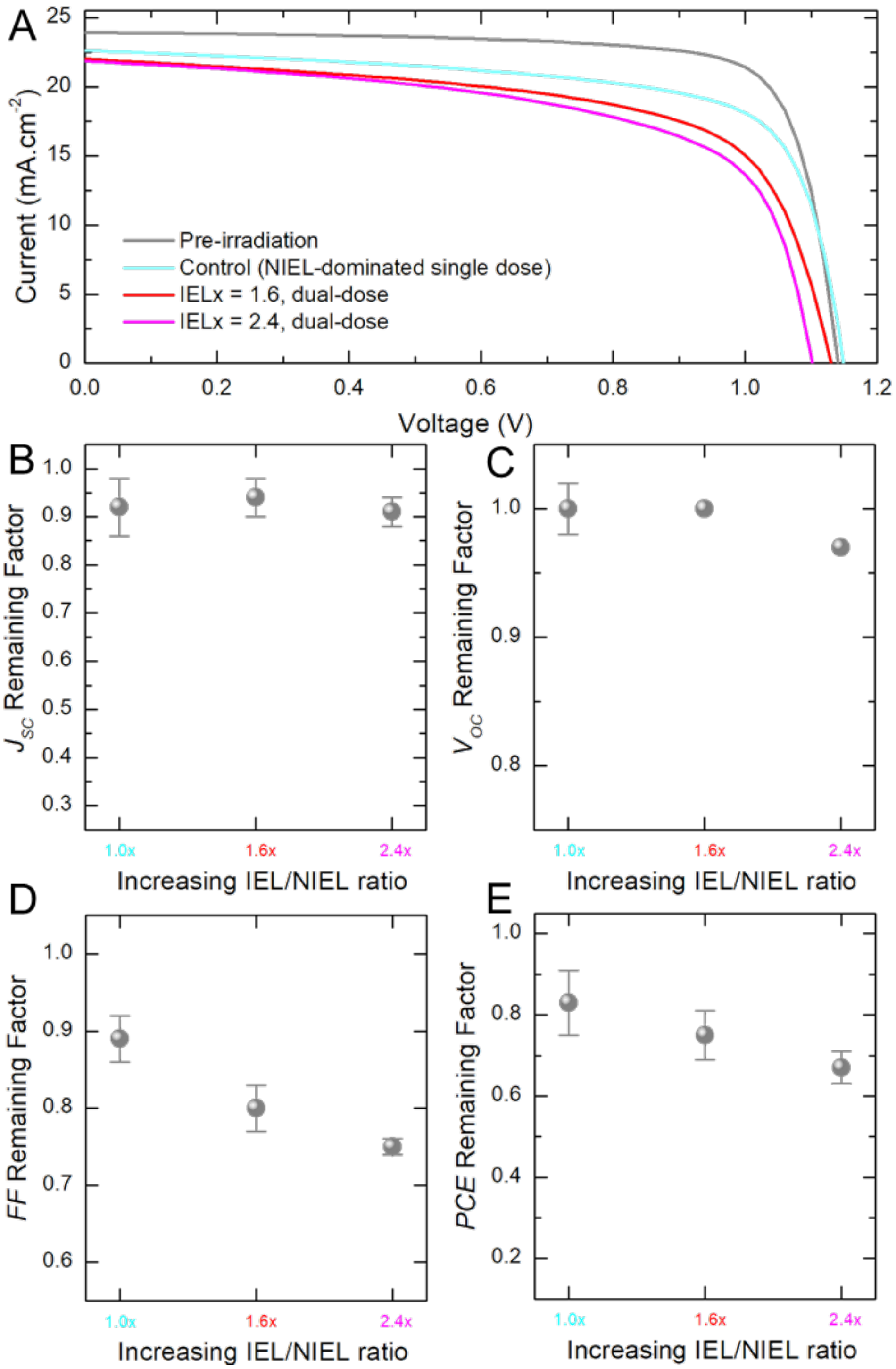

Supplementary Figure 18. Dual-dose experiments on device architecture employing a modified charge transport layer.

A. *J*-*V* curves of representative higher PCE *PIN* solar cells prior to irradiation (grey), after irradiation with the NIEL-dominated single dose of 0.06 MeV protons (cyan), and after being healed with dual dose radiation (red, magenta). B. J_{SC}, C. V_{OC}, D. *FF*, and E. *PCE* remaining

factors are also shown, and parameters are averaged over 4-5 devices. Error bars represent standard deviation.

Supplementary Table 2.

Summary of higher PCE *PIN* device parameters for various irradiation conditions. Parameters are averaged over 4-5 devices. Error bars represent standard deviation.

Device	Rad. 1 (MeV, cm^{-2})	Rad. 2 (MeV, cm^{-2})	IEL x	J_{SC} ($mA.cm^{-2}$)	V_{OC} (Volts)	*FF*	*PCE* (%)	PCE Remaining factor
1.	0.06, 1×10^{13}	-	-	23.99 ± 0.31	1.13 ± 0.01	0.79 ± 0.00	21.46 ± 0.89	0.83 ± 0.08
				22.21 ± 1.31	1.13 ± 0.02	0.71 ± 0.02	17.84 ± 1.53	
2.	0.06, 0.97×10^{13}	1.0, 1.13×10^{13}	1.6	23.72 ± 0.35	1.13 ± 0.00	0.79 ± 0.01	21.32 ± 0.68	0.75 ± 0.06
				22.31 ± 0.83	1.13 ± 0.00	0.63 ± 0.02	15.99 ± 1.12	
3.	0.06, 0.93×10^{13}	1.0, 2.64×10^{13}	2.4	24.11 ± 0.21	1.13 ± 0.00	0.80 ± 0.01	21.92 ± 0.46	0.67 ± 0.04
				22.07 ± 0.60	1.10 ± 0.00	0.60 ± 0.01	14.64 ± 0.74	

Supplementary Note 1.

These $Cs_{0.05}(FA_{0.92}MA_{0.08})_{0.95}Pb(I_{0.92}Br_{0.08})_3$ *PIN* PSCs were fabricated according to the hole-selective contact and perovskite absorber co-deposition method reported recently.[3] The hole-selective contact and perovskite absorber were cast in a single coating step in a N_2 glove box. 1.4 M perovskite precursor containing 0.5 mg/ml Me-4PACz was spin-coated onto UV-ozone-treated ITO substrates at 2,000 r.p.m. for 2 s and 4,000 r.p.m. for 20 s, and then 150 µl CB was dropped onto the spinning substrate 5 s before the end of the spin-coating process. Subsequently, the sample was annealed at 100 °C for 30 min. The devices were completed by sequentially thermally evaporating LiF (1 nm), C_{60} (25 nm), BCP (6 nm) and silver (100 nm).

As the **Supplementary Fig. 18** and **Supplementary Table 2** above show, the NIEL-dominated single dose irradiation (0.06 MeV protons) was unable to create the same level of damage in these higher PCE cells resulting in higher remaining factors of 0.83 ± 0.08. Given the low density of defects after this irradiation, these devices do not require healing and dual dose irradiation leads to a decrease in the remaining factor. While the differences in fabrication method and device behavior are too substantial to compare directly and can be a separate in-depth study, these data

highlight the subtle link between the initial defect density created by the NIEL-dominated radiation and IEL-induced healing.

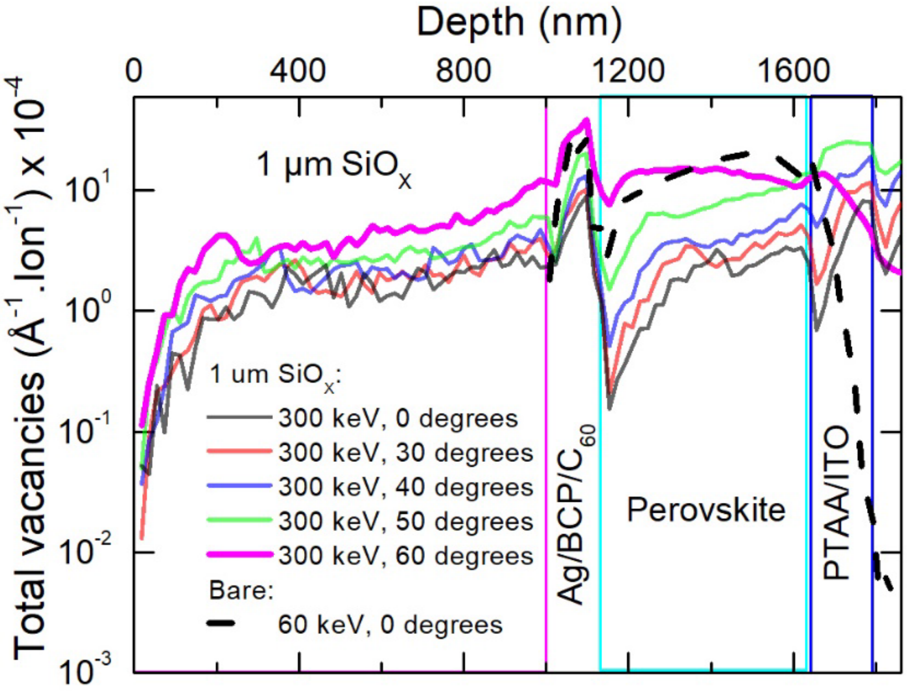

Supplementary Figure 19: SRIM vacancy profiles for 1 µm SiO$_X$ PIN PSCs.
Vacancy profiles in SiO$_X$ PSCs for 300 keV protons incident at various angles simulated using SRIM. The control case of 60 keV protons normally incident on a bare cell is also shown.

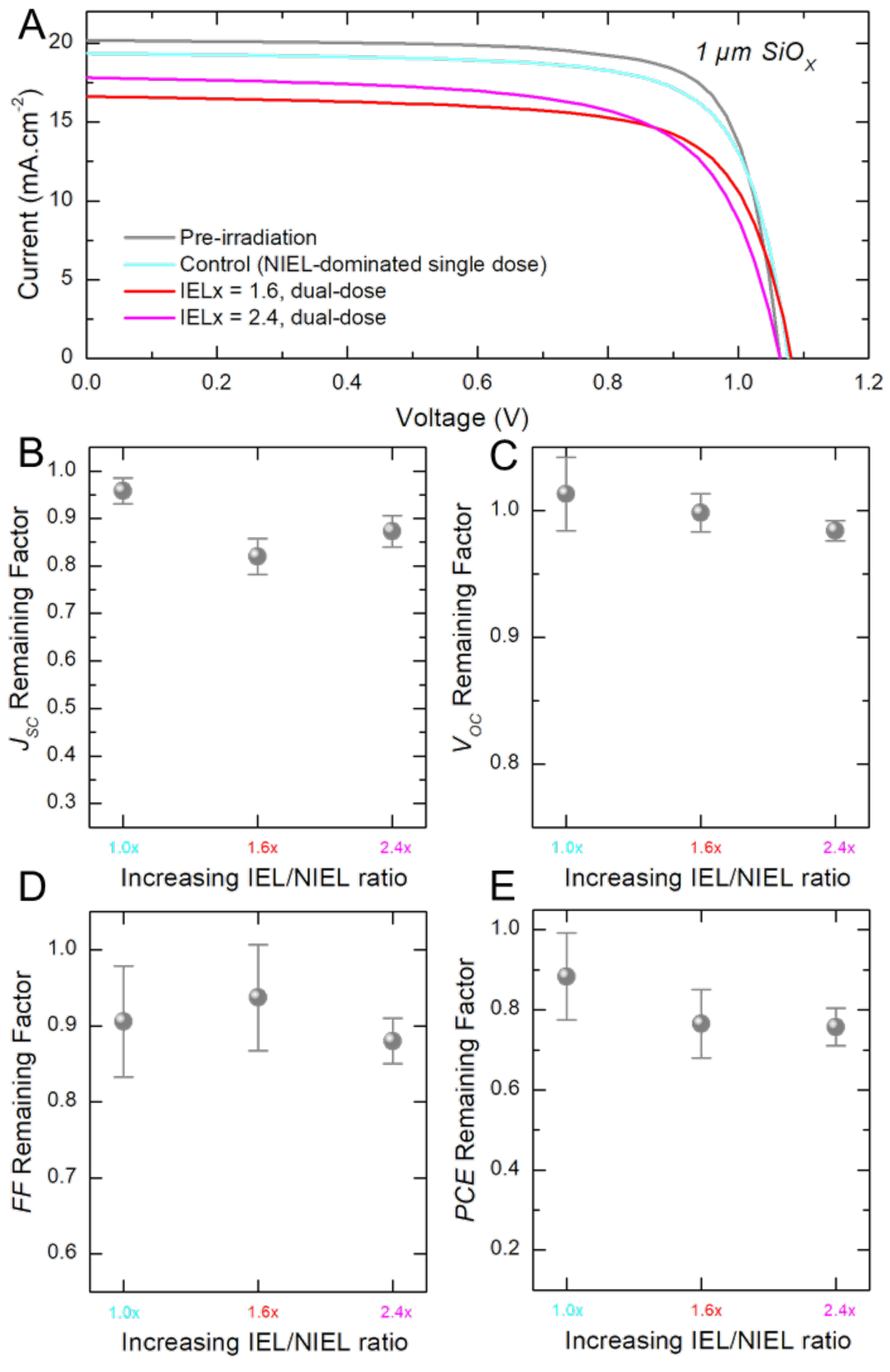

Supplementary Figure 20: Dual-dose experiments on 1 µm-SiO_X *PIN* PSCs.

A. *J-V* curves of representative solar cells prior to irradiation (grey), after irradiation with the NIEL-dominated single dose of 0.06 MeV protons (cyan), and after dual dose irradiation (red, magenta). Remaining factors for B. J_{SC}, C. V_{OC}, D. *FF*, and E. *PCE* for the irradiated PSCs for IEL x = 1.6, 2.4 scenarios. The single dose control device is denoted by the IEL x = 1.0 case. Parameters are averaged over 5 devices. Error bars represent standard deviation.

Supplementary Note 2.

Next, we considered solar cells encapsulated with our recently-demonstrated 1 μm thick silicon oxide (SiO_X) barrier layer. The barriers were deposited as per our recent report. These barriers have been shown to block the harmful low-energy protons while reducing the deleterious impact of fully-penetrating protons and alpha particles likely by serving as a chemical barrier to effusion of organic and gaseous products from the device stack upon irradiation. SRIM pointed us to the adequate proton energies for carrying out dual dose experiments on the SiO_X cells.

We carried out SRIM simulations to understand as to what proton energies would be adequate for the SiO_X cells to create the damage profiles created in a bare cell without SiO_X. Based on our earlier papers,[2,4] 1 μm SiO_X will absorb 0.105 MeV energy from the incident proton. In other words, the incident energies of 0.06 MeV and 1.00 MeV should be adequately increased to 0.165 MeV and 1.105 MeV for carrying out these experiments on SiO_X cells. However, our accelerator cannot generate protons in the energy range of 0.1 – 0.3 MeV. We therefore sought a way to use 0.3 MeV protons for the single dose irradiation. It is clear from **Supplementary Fig. 19** that the vacancy profiles for normally incident 0.06 MeV and 0.3 MeV protons are vastly different. Tuning the incident angle for 0.3 MeV protons reveals that 60° incidence of these protons on the SiO_X device can result in a vacancy profile similar to that created by 0.06 MeV protons normally incident on a bare device.

Supplementary Fig. 20 shows *J-V* curves and remaining factors for solar cells exposed to the NIEL-dominated single dose condition of 0.3 MeV (60°) and the dual dose conditions involving 0.3 MeV protons (60°) and 1.105 MeV protons. The single dose irradiation (0.3 MeV protons) was not able to create sufficient damage in these protected cells, resulting in high remaining factors approaching 0.90. Given a significantly low radiation-induced defect density, healing was not observed for the dual dose irradiation conditions used.

It is also possible that the slight differences in the vacancy profiles between the 0.3 MeV (60°) protons on the SiO_X cell and normally incident 0.06 MeV protons on a bare cell prohibited observation of the healing effect (Supplementary Fig. 19). This highlights the complications associated with the SiO_X cell irradiation, while also demonstrating the radiation protection benefits of SiO_X.

Finally, it is possible that these device architectures (Supplementary Fig. 18, Supplementary Fig. 20) have better local thermal management that allows for instantaneous temperature dissipation leading to healing even for NIEL-dominated irradiation. Though outside the scope of this work, phonon transport engineering at the nanoscale can be an effective strategy for mitigating radiation-induced damage in semiconductors with strong electron-phonon coupling such as metal-halide perovskites.

Supplementary References

1. https://sr-niel.org/.
2. Kirmani, A. R. *et al.* Countdown to perovskite space launch: Guidelines to performing relevant radiation-hardness experiments. *Joule* **6**, 1015-1031, doi:10.1016/j.joule.2022.03.004 (2022).
3. Zheng, X. *et al.* Co-deposition of hole-selective contact and absorber for improving the processability of perovskite solar cells. *Nature Energy* **8**, 462-472, doi:10.1038/s41560-023-01227-6 (2023).
4. Kirmani, A. R. *et al.* Metal oxide barrier layers for terrestrial and space perovskite photovoltaics. *Nature Energy* **8**, 191-202, doi:10.1038/s41560-022-01189-1 (2023).
