# Supplementary material for: Unraveling radiation damage and healing mechanisms in halide perovskites using energy-tuned dual irradiation dosing
Source: Nat Commun. 2024 Jan 24;15:696. doi: 10.1038/s41467-024-44876-1 (PMC10810841; doi:10.1038/s41467-024-44876-1)
Supplement: Supplementary file 3 — Reporting Summary [file 41467_2024_44876_MOESM3_ESM.pdf]

nature research

Corresponding author(s): Joseph M. Luther, Ahmad R. Kirmani

Solar Cells Reporting Summary

Nature Research wishes to improve the reproducibility of the work that we publish. This form is intended for publication with all accepted papers reporting the characterization of photovoltaic devices and provides structure for consistency and transparency in reporting. Some list items might not apply to an individual manuscript, but all fields must be completed for clarity.

For further information on Nature Research policies, including our data availability policy, see Authors & Referees.

Experimental design

Please check: are the following details reported in the manuscript?

1. Dimensions

Item	Yes	No	Explanation
Area of the tested solar cells	☒	☐	'J-V characterization' sub-section under 'Methods' section.
Method used to determine the device area	☒	☐	'J-V characterization' sub-section under 'Methods' section.

2. Current-voltage characterization

Item	Yes	No	Explanation
Current density-voltage (J-V) plots in both forward and backward direction	☒	☐	'J-V characterization' sub-section under 'Methods' section.
Voltage scan conditions<br>*For instance: scan direction, speed, dwell times*	☒	☐	'J-V characterization' sub-section under 'Methods' section.
Test environment<br>*For instance: characterization temperature, in air or in glove box*	☒	☐	'J-V characterization' sub-section under 'Methods' section.
Protocol for preconditioning of the device before its characterization	☐	☒	Preconditioning not needed for these devices
Stability of the J-V characteristic<br>*Verified with time evolution of the maximum power point or with the photocurrent at maximum power point; see ref. 7 for details.*	☐	☒	Stability and high efficiency are not the main focus of this paper

3. Hysteresis or any other unusual behaviour

Item	Yes	No	Explanation
Description of the unusual behaviour observed during the characterization	☐	☒	Devices didn't exhibit any unusual behavior during characterization
Related experimental data	☐	☒	Devices didn't exhibit any unusual behavior during characterization

4. Efficiency

Item	Yes	No	Explanation
External quantum efficiency (EQE) or incident photons to current efficiency (IPCE)	☐	☒	EQE data was not needed as Jsc is not a focus of this paper
A comparison between the integrated response under the standard reference spectrum and the response measure under the simulator	☐	☒	Record devices with high Jsc (current) values are not a focus of this paper
For tandem solar cells, the bias illumination and bias voltage used for each subcell	☐	☒	This paper does not report any tandem solar cells

5. Calibration

Item	Yes	No	Explanation
Light source and reference cell or sensor used for the characterization	☒	☐	'J-V characterization' sub-section under 'Methods' section.
Confirmation that the reference cell was calibrated and certified	☒	☐	'J-V characterization' sub-section under 'Methods' section.

November 2017

Calculation of spectral mismatch between the reference cell and the devices under test

☒ Yes ☐ No — 'J-V characterization' sub-section under 'Methods' section.

6. Mask/aperture

Size of the mask/aperture used during testing

☒ Yes ☐ No — 'J-V characterization' sub-section under 'Methods' section.

Variation of the measured short-circuit current density with the mask/aperture area

☐ Yes ☒ No — Device performance as a function of mask area is not the focus of this paper

7. Performance certification

Identity of the independent certification laboratory that confirmed the photovoltaic performance

☐ Yes ☒ No — This paper does not report record solar cell efficiencies

A copy of any certificate(s)
Provide in Supplementary Information

☐ Yes ☒ No — This paper does not report record solar cell efficiencies

8. Statistics

Number of solar cells tested

☒ Yes ☐ No — Captions of Figures 2, 4, Table 1, 2, Supplementary Figures 6, 15, 16, 18, 20, Supplementary Table 2

Statistical analysis of the device performance

☒ Yes ☐ No — Table 1, Table 2, Supplementary Table 2

9. Long-term stability analysis

Type of analysis, bias conditions and environmental conditions
For instance: illumination type, temperature, atmosphere humidity, encapsulation method, preconditioning temperature

☐ Yes ☒ No — Stability and high efficiency are not the main focus of this paper
